# Spatial Profiling and Prognostic Role of CD169+ Macrophages in Colorectal Cancer From Adjacent Nontumor Mucosa to Liver Metastasis

**DOI:** 10.1155/jimr/7305785

**Published:** 2026-09-29

**Authors:** Wenjing Ye, Sergii Pavlov, Esraa Ali, Filip Ambrozkiewicz, Lenka Červenková, Ondřej Vyčítal, Petr Hošek, Ondřej Daum, Václav Liška, Kari Hemminki, Andriy Trailin

**Affiliations:** ^1^ Laboratory of Translational Cancer Genomics, Biomedical Center, Faculty of Medicine in Pilsen, Charles University, Alej Svobody 1665/76, 32300, Pilsen, Czech Republic, cuni.cz; ^2^ Laboratory of Cancer Treatment and Tissue Regeneration, Biomedical Center, Faculty of Medicine in Pilsen, Charles University, Alej Svobody 1665/76, 32300, Pilsen, Czech Republic, cuni.cz; ^3^ Department of Surgery, University Hospital and Faculty of Medicine in Pilsen, Charles University, Alej Svobody 80, 32300, Pilsen, Czech Republic, cuni.cz; ^4^ The Department of Pathology, Regional Hospital Liberec, Liberec 46001, Czech Republic; ^5^ Department of Cancer Epidemiology, German Cancer Research Center, Im Neuenheimer Feld 280, 69120, Heidelberg, Germany, dkfz.de

**Keywords:** CD169, colorectal cancer, liver metastases, macrophages, metachronous, survival analysis, synchronous

## Abstract

**Background:**

CD169+ cells represent a distinct subset of macrophages with potential immunomodulatory roles, but their function in colorectal cancer (CRC) progression remains unclear. This study focuses on the spatial profiling and prognostic significance of CD169+ macrophages in adjacent nontumor mucosa (NM), primary CRC (pCRC), and synchronous or metachronous liver metastases (LM).

**Materials and Methods:**

We enrolled into this retrospective cohort study patients who underwent resection of both pCRC with adjacent NM and synchronous LM (*N* = 55) or metachronous LM (*N* = 44). We applied immunohistochemical staining and computer‐assisted image analysis to quantify CD169+ cell density in the NM, pCRC, synchronous, and metachronous LM. Correlations between CD169+ macrophages and T cells were evaluated. Associations between CD169+ cell density and overall survival (OS) of the patients were assessed.

**Results:**

CD169+ cell density was greater in NM than in the tumor center (TC) of pCRC and in the peritumor (PT) region of LM compared to pCRC. The synchronous group exhibited a higher density of CD169+ cells than the metachronous group in the inner margin (IM) of pCRC, the TC, and the outer margin (OM) of LM. CD169+ cells were more abundant in the exterior than in the interior tumor areas in both pCRC and LM. Positive correlations were observed between densities of CD169+ macrophages with CD3+ and CD8+ lymphocytes. In the synchronous group, a high density of CD169+ macrophages in the TC of pCRC was associated with longer OS, whereas a high density in the PT of LM was associated with shorter OS. High density of CD169+ macrophages in the OM of metachronous LM was associated with poor survival.

**Conclusion:**

Our study highlights context‐dependent prognostic associations of CD169+ macrophages in CRC. While high CD169+ macrophage density in the TC of pCRC was linked to favorable survival in the synchronous group, increased density in both synchronous and metachronous LM was associated with adverse outcomes. Further prospective studies and functional investigations are needed to validate these observations.

## 1. Introduction

Colorectal cancer (CRC) is the third most commonly diagnosed cancer with a high mortality rate worldwide [1]. The overall 5‐year survival rate for patients with CRC is 50%–65%, and individual prognosis depends largely on whether they develop metastases during the course of the disease [2]. Despite the prognosis of patients with CRC having improved over the past decades due to early screening [3], 15%–25% present with synchronous liver metastases (LM), and up to 30% of patients at stages I–III develop metachronous LM later [4–6]. Synchronous and metachronous LM have been reported to represent biologically and clinicopathologically distinct entities [7, 8]. However, the immune microenvironment of synchronous and metachronous LM has been rarely compared before and requires further investigation.

The tumor microenvironment (TME) exhibits diverse capabilities that can either support or suppress tumor development, depending on its composition and context [8]. Tumor‐associated macrophages (TAMs) in the TME constitute the predominant immune cell population within the TME [9]. Macrophage anticancer responses are triggered by the recognition of cancer cells, primarily through TLR‐mediated detection of danger‐associated molecular patterns, especially via TLR4 [10–12]. Characterized by high phenotypic and functional heterogeneity, TAMs play a pivotal role in the development of both primary and metastatic tumors [11, 13, 14]. Different macrophage subtypes shape CRC progression from early adenoma to liver metastasis and predominate across distinct consensus molecular subtypes of CRC [11, 15].

CD169+ macrophages are a unique subset that cannot be categorized strictly as M1 (classically activated, proinflammatory) or M2 (alternatively activated, immunoregulatory) macrophages [15]. Predominantly located in secondary lymphoid organs, they play specialized roles in antigen presentation, immune regulation, and antiviral responses—functions that extend beyond the conventional M1/M2 paradigm [16, 17]. Notably, several studies have reported that CD169+ macrophages can simultaneously express proinflammatory cytokines such as IL‐6 and TNF‐α, alongside the antiinflammatory cytokine IL‐10 [18, 19]. Furthermore, in peripheral blood and liver tissue from patients with cirrhosis, CD169+ macrophages were shown to coexpress markers of both M1‐like (HLA‐DR and CD86) and M2‐like (CD206) phenotypes [19], suggesting a highly plastic phenotype that is responsive to environmental cues.

In the colon mucosa, CD169+ macrophages act as sentinels and amplifiers of inflammation during injury or colitis by promoting Th17 responses and recruiting inflammatory monocytes through CCL8 secretion [20, 21]. Notably, dysbiosis has been identified as a potential upstream trigger of CRC [12, 22], wherein disruption of the host–microbiota interface drives aberrant TLR/MyD88 activation and transitions of CD169+ macrophages toward a proinflammatory phenotype [21, 23, 24].

Previous studies have shown that CD169+ macrophages in the regional lymph nodes of CRC patients mediate antigen presentation and activate CD8+ T cells, contributing to a favorable prognosis [25, 26]. In contrast, other research has reported that CD169+ cells in the peripheral blood of CRC patients are associated with IL‐10 production and tumor progression [18]. Despite these findings, it remains unknown how to investigate the spatial distribution and prognostic significance of CD169+ macrophages across adjacent nontumor mucosa (NM), primary tumor (primary CRC [pCRC]), and LM in CRC.

Our study thus aims at elucidating the distribution, clinicopathological associations, and prognostic significance of CD169+ macrophages across pCRC, NM, and LM in patients with synchronous and metachronous disease.

## 2. Materials and Methods

### 2.1. Patient Cohorts

This retrospective study included patients from the Pilsen University Hospital, who were diagnosed with stage I–IV CRC and received surgery for both primary tumor and either synchronous or metachronous LM during 1999–2023. The study was approved by the Ethics Committee of the Faculty of Medicine and the University Hospital in Pilsen (300/2020, June 17, 2020).

Synchronous LM was defined as metastases diagnosed prior to or concurrently with the pCRC diagnosis, including lesions discovered during primary tumor resection. Metachronous LM was defined as metastases diagnosed following the initial staging and surgical treatment of the pCRC [27].

Paired formalin‐fixed paraffin‐embedded (FFPE) specimens of pCRC, LM, and NM were obtained retrospectively from two cohorts: stage IV patients (*n* = 80) presenting with synchronous LM at primary tumor surgery and stage I–III patients (*n* = 100) who experienced metachronous LM occurrence within 1–59 months after resection of pCRC (median, 17 months).

Exclusion criteria: Patients did not have paired triplicate samples of NM, pCRC, and LM; insufficient quality of FFPE tissue or sections; whole slide images with poor quality that interfere with analysis; missing clinical data; and incomplete follow‐up.

After exclusion, 99 patients were enrolled into the study, including 55 with synchronous LM and 44 with metachronous LM.

The clinical endpoint of this study was overall survival (OS), the time from the resection of LM to the date of death of any cause.

### 2.2. Immunohistochemistry

About 4 μm sections from each FFPE block were stained immunohistochemically for CD169 with primary monoclonal antibodies from Abcam, clone SP213 (Abcam Limited, Cambridge, UK). The detailed process of staining can be found in one of our previous studies [28]. The stained sections were then digitized into whole slide images using an Olympus VS200 scanner (Shinjuku, Japan) at 20× magnification. Negative and positive (tonsils) controls were used throughout. All immunohistochemical staining was performed using standardized protocols under identical experimental conditions.

### 2.3. Quantification of Macrophages

Image preprocessing steps were performed using Qupath (v 0.5.1) software. Full details of QuPath, including source code, documentation, and links to the software download, are available at https://qupath.github.io. A predefined script was applied to all pCRC and LM images based on tumor nest annotations, which automatically segmented four distinct regions of interest (ROIs): tumor regions were first annotated along the interface between malignant cell nests and adjacent nontumor tissue. The inner margin (IM) and outer margin (OM) were extended automatically as 500 μm‐wide regions on each side of the border. The tumor center (TC) represents the remaining tumor area. The peritumor (PT) area was defined as the 500 μm‐wide regions adjacent to the OM [28]. NM was annotated manually, including all the epithelium, crypts, and lamina propria, while muscularis mucosa was excluded. A trained classifier in QuPath, using automated algorithms, was employed to distinguish tissue from the background. Areas of artifacts (tissue folding, bubbles, necrosis, abscesses, mucin pools, and large vessels) were excluded manually. CD169+ cells were quantified using the “positive cell detection” tool with identical detection parameters for all slides to ensure reproducibility. QuPath data output included numbers of CD169+ cells per mm^2^ within each ROI (cell density).

### 2.4. Follow‐up

Clinical follow‐up was performed in accordance with current guidelines and is available until December 2023. The median follow‐up after liver metastasectomy was 84 months in the synchronous group and 61 months in the metachronous group.

### 2.5. Outcomes

The primary outcome was OS, defined as the interval from LM resection to death from any cause. Patients alive at the end of follow‐up were censored at their last recorded visit. By using LM resection as time zero, we specifically assessed the prognostic value of CD169+ macrophages in the synchronous vs. metachronous group while minimizing a lead‐time bias introduced by differences in the timing of metastasis development [29].

### 2.6. Statistical Analysis

Nonnormally distributed data are expressed as median (min–max); their comparison was made by Mann–Whitney *U*‐test for independent variables; for dependent variables, we used Friedman ANOVA with Dunn’s multiple comparison test or the Wilcoxon matched‐pairs test, as appropriate. The associations between macrophage cell density in pCRC and LM as well as between macrophages and T cells (CD3+ and CD8+ cells), were assessed using Spearman’s correlation. The densities of CD3+ and CD8+ cells were obtained from our earlier study on the same cohort [28].

For survival analysis, to eliminate skewness in the distribution, raw cell density in each ROI was converted into percentiles and categorized into low (<25th percentile) and high (25th–100th percentile) groups. We chose the 25th‐percentile cutoff to identify a biologically meaningful low‐density subgroup. This prespecified, reproducible threshold ensures balanced group size and reliable prognostic stratification while minimizing post‐hoc overfitting [28, 30]. Continuous‐variable models were also tested. OS was estimated using the Kaplan–Meier method and compared between groups with the log‐rank test, with statistical significance defined as *p* < 0.05. Hazard ratios (HRs) with 95% confidence intervals (CIs) were calculated using the Cox model. Univariable Cox regression was followed by multivariable analysis using confounders, which were either significantly different between groups or significantly predictive for OS. Statistical analysis was carried out using R language packages (such as survival [31], finalfit [32], and survminer [33]) and GraphPad Prism 8.0. To evaluate result robustness, we calculated bootstrapped 95% CIs for OS HRs across both continuous and dichotomous variables. The bootstrapping was performed in Matlab (v2023b) using the bias‐corrected and accelerated percentile method with 5000 resamples for continuous variables and 20,000 for dichotomous variables.

To account for multiple testing (e.g., cell density was assessed in 9 ROIs per patient), the false discovery rate (FDR) was controlled across all study analyses using the Benjamini–Hochberg procedure. At a conventional *α* = 0.05 threshold, the estimated FDR is 14.5%, implying that ~85.5% of significant findings are expected to be true positives. Achieving a stricter overall FDR of 5% would necessitate an individual test significance threshold of *α* = 0.01.

## 3. Results

### 3.1. Patient Outcomes

The 3‐year OS rate was 51.9% in the synchronous group and 62.6% in the metachronous group (Table S1). During follow‐up, death events were observed in 42 (76.4%) patients with synchronous LM and 24 (54.5%) patients with metachronous LM.

### 3.2. Clinical and Pathological Characteristics of Patients

Compared to the synchronous group, the metachronous group exhibited significantly larger LM, fewer patients treated with FOLFOX, and a lower rate of chemotherapy before liver resection (Table 1).

**Table 1:** Demographics and clinicopathological features of patients.

Parameter			Synchronous (*N* = 55)	Metachronous (*N* = 44)	*p*
Age at the diagnosis (years), median (min–max)			62 (29–78)	64 (46–73)	0.31
Gender		Male	34 (61.8%)	29 (65.9%)	0.67
		Female	21 (38.2%)	15 (34.1%)	
Primary tumor					
Location		Right colon	12 (21.8%)	8 (18.2%)	0.65
		Left colon	43 (78.2%)	36 (81.8%)	
Size (cm), median (min–max)			4.3 (1.0–7.5)	3.5 (0.8–8.3)	0.06
Pathologic T stage (pT)		T1	0 (0.0%)	1 (2.3%)	0.25
		T2	1 (1.8%)	6 (13.6%)	
		T3	48 (87.3%)	35 (79.5%)	
		T4	6 (10.9%)	2 (4.5%)	
Histological type		NOS	51 (92.7%)	39 (88.6%)	
		Mucinous	2 (3.6%)	4 (9.1%)	
		Other	2 (3.6%)	1 (2.3%)	
Pathologic N stage		N0	13 (23.6%)	13 (29.5%)	0.51
		N1	22 (40.0%)	16 (36.4%)	
		N2	20 (36.4%)	15 (34.1%)	
AJCC 8th staging		Stage I		1 (2.3%)	
		Stage II		12 (27.3%)	
		Stage III		31 (70.4%)	
		Stage IV	55 (100%)		
Grade		1	12 (21.8%)	14 (31.8%)	0.77
		2	37 (67.3%)	26 (59.1%)	
		3	6 (10.9%)	4 (9.1%)	
KRAS status		Mutated	20 (36.4%)	11 (25.0%)	0.26
		WT	27 (49.1%)	25 (56.8%)	
		Not tested	8 (14.5%)	8 (18.2%)	
BRAF status		Mutated	0 (0.0%)	1 (2.3%)	0.27
		WT	44 (80.0%)	35 (79.5%)	
		Not tested	11 (20.0%)	8 (18.2%)	
MSI status		High	1 (1.8%)	1 (2.3%)	0.9
		Low	42 (76.4%)	35 (79.5%)	
		Not tested	12 (21.8%)	8 (2.3%)	
CEA ng/mL			6.8 (1.0–945.0)	8.9 (0.8–1649.0)	0.9
CEA		<5 ng/mL	18 (46.2%)	13 (46.4%)	
		>5 ng/mL	21 (53.8%)	15 (53.6%)	0.98
Number of examined lymph nodes			13 (1–34)	11 (0–43)	0.38
Lymph node ratio, median (25–75 percentile)			0.22 (0–0.57)	0.13 (0–0.32)	0.14
Liver metastases					
Number, median (min–max)			2 (1–24)	1 (1–7)	0.08
Size (cm), median (min–max)			2.0 (0.4–26.0)	2.7 (0.6–7.1)	**0.02**
Grade		1	21 (39.6%)	18 (42.9%)	0.75
		2	32 (60.4%)	24 (57.1%)	
Surgical margin status for metastasectomy		R0	40 (76.9%)	29 (69.0%)	0.39
		R1	12 (23.1%)	13 (31.0%)	
Chemotherapy ± targeted therapy		CHT alone	28 (50.9%)	31 (70.5%)	0.07
		CHT + anti‐VEGF	11 (20.0%)	4 (9.1%)	0.06
		CHT + anti‐EGFR	9 (16.4%)	6 (13.6%)	
		No treatment	4 (7.3%)	2 (4.5%)	
		Not known	3 (5.4%)	3 (6.8%)	
Chemotherapy regimens		FOLFOX	31 (56.4%)	16 (36.4%)	**0.05**
		de Gramont	7 (12.7%)	9 (20.5%)	
		FOLFIRI	4 (7.3%)	2 (4.5%)	
		Others	6 (10.9%)	12 (27.3%)	
Timing of chemotherapy		Before liver surgery	37 (77.1%)	11 (26.8%)	**0.0001**
		After liver surgery	11 (22.9%)	30 (73.2%)	

*Note*: Bold values indicate statistical significance at the *p* < 0.05 level.

Abbreviations: AJCC, The American Joint Committee on Cancer; CEA, the carcinoembryonic antigen; CHT, chemotherapy; MSI, microsatellite instability; NOS, not otherwise specified; R0, no tumor cells detected at the resection margin; R1, tumor cells present at the microscopic resection margin; WT, wild type.

### 3.3. Morphology and Distribution of CD169+ Macrophages in NM, pCRC, and LM

CD169+ macrophages display a distinct membranous staining pattern throughout the three tissues. A majority of CD169+ macrophages in the NM were observed in lamina propria and predominated in the superficial half of the mucosa. In mucosa‐associated lymphoid follicles, CD169+ macrophages were scarce, if present at all, situated around follicles and in the mantle and marginal zones of secondary follicles (Figure 1A). In NM, cells had diverse morphology from rounded to spindle‐ or stellate‐shaped due to frequent surface protrusions or pseudopodia (Figure 1B).

**Figure 1:**
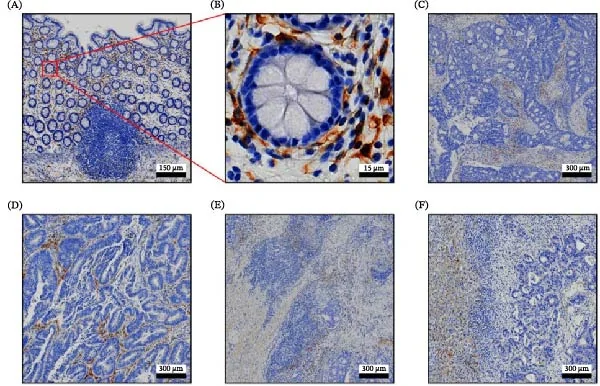
Representative images from the analyzed cohort showing distribution and morphology of CD169+ macrophages across NM, pCRC, and LM following immunoperoxidase staining. (A) NM tissue; (B) morphology of CD169+ macrophages in NM, with the corresponding region indicated in panel (A); (C) tumor center of pCRC; (D) tumor center of LM; (E) tumor‐invasive margin of pCRC; and (F) tumor‐invasive margin of LM. Similar staining patterns were observed across the analyzed specimens. Abbreviations: LM, liver metastasis; NM, nontumor mucosa; pCRC, primary colorectal cancer.

The vast majority of CD169+ cells in pCRC and LM had a stromal location and exhibited a predominantly elongated or spindle shape. These cells were mainly distributed around the tumor‐invasive front, with few being presented in the TC (Figure 1C–E). In the PT liver, CD169+ cells were primarily located in the perivascular region along blood and lymphatic capillaries and distributed along hepatic sinusoids (Figure 1F). Notably, lymphoid aggregates around pCRC and LM showed great variability in the numbers of CD169+ cells, from none to moderate.

### 3.4. Density of CD169+ Macrophages in NM, pCRC, and LM

After comparing the density of CD169+ cells between NM and pCRC in the synchronous and metachronous groups, greater cell density was found in NM vs. the TC of pCRC (Figure 2A) in both groups. When compared between pCRC and LM, only the PT region of synchronous LM showed significantly greater CD169+ macrophage cell density than the PT region of the primary tumor (Figure 2B).

**Figure 2:**
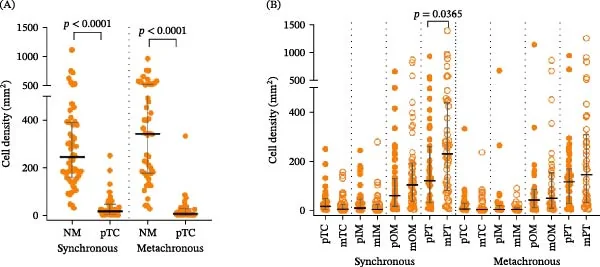
Distribution of CD169+ cell density in NM, pCRC, and LM. (A) Cell density between NM and pTC in the synchronous group (*n* = 53) and metachronous group (*n* = 42). (B) Cell density between ROIs of pCRC and LM in the synchronous group (*n* = 51) and metachronous group (*n* = 40). Statistical significance was determined using the Wilcoxon matched‐pairs signed‐rank test. Black lines represent medians; gray error bars represent interquartile range. Abbreviations: LM, liver metastasis; mOM, outer margin of LM; mPT, peritumor area of LM; mTC, tumor center of LM; medians, r margin of LM; NM, nontumor mucosa; pCRC, primary colorectal cancer; pIM, inner margin of pCRC; pOM, outer margin of pCRC; pPT, peritumor area of pCRC; pTC, tumor center of pCRC; ROIs, regions of interest.

For interregion comparisons, the PT region and OM showed significantly greater CD169+ macrophage cell density vs. TC and IM in both pCRC and LM in the synchronous and metachronous groups (Figure 3A, B).

**Figure 3:**
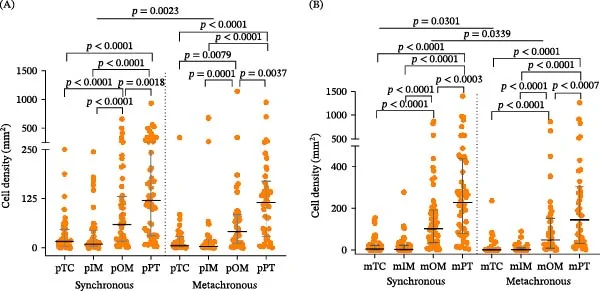
Distributions of CD169+ cell density in different ROIs of (A) pCRC in the synchronous group (*n* = 54) and metachronous group (*n* = 42), and (B) LM in the synchronous group (*n* = 52) and metachronous group (*n* = 42). Statistical significance was determined using Friedman and Dunn’s multiple comparisons test in this figure. Black lines represent medians; gray error bars represent interquartile range. Abbreviations: LM, liver metastasis; mIM, inner margin of LM; mOM, outer margin of LM; mPT, peritumor area of LM; mTC, tumor center of LM; pCRC, primary colorectal cancer; pIM, inner margin of pCRC; pOM, outer margin of pCRC; pPT, peritumor area of pCRC; pTC, tumor center of pCRC; ROIs, regions of interest.

We also found greater density of CD169+ cells in the IM of pCRC (Figure 3A) and also in both TC and OM of LM (Figure 3B) in the synchronous over the metachronous group.

### 3.5. Correlations of CD169+ Cells Between NM and pCRC and between pCRC and LM

We did not observe any correlation of CD169+ cell density between NM and pCRC, as well as between pCRC and LM in the synchronous group or in the metachronous group (data not shown).

### 3.6. Correlations of CD169+ Cells and T Cells

Density of CD169+ macrophages correlated positively with CD3+ T cells in the PT of pCRC, TC, and IM of LM only in the synchronous group (Table S2). CD169+ cell density correlated positively with CD8+ T cells in the TC and IM of pCRC in the metachronous group and in the TC and IM of LM in the synchronous group (Table S3).

### 3.7. Associations Between CD169+ Macrophage Density and Survival

CD169+ macrophage density in NM was not associated with any of the patient outcomes (results are not shown).

In the synchronous group, a high density of CD169+ macrophages in the TC of pCRC was significantly associated with a longer OS (Table 2), whereas a high density in the PT of LM was associated with a shorter OS with borderline statistical significance. High CD169+ macrophage density in the OM of metachronous LM showed a negative association with OS (Table 2). Similar associations were also observed in Kaplan–Meier analysis (Figure 4A, B). When evaluated as continuous variables, only CD169+ macrophage density in the PT of synchronous LM demonstrated a significant association with a shorter OS (Table S4).

**Figure 4:**
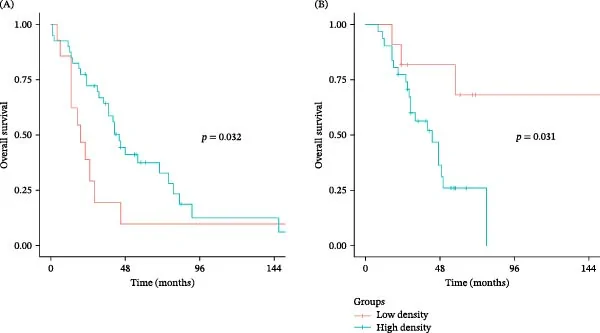
Kaplan–Meier survival curves showing associations between overall survival with high vs. low density of CD169+ cells in ROIs of pCRC and LM. (A) TC of pCRC in synchronous group, low density, *n* = 14; high density, *n* = 40, *p* = 0.034. (B) OM of LM in the metachronous group, low density, *n* = 11; high density, *n* = 31, *p* = 0.042. *p*‐Values are according to the log‐rank test. Abbreviations: LM, liver metastasis; OM, outer margin; pCRC, primary colorectal cancer; ROIs, regions of interest; TC, tumor center.

**Table 2:** Hazard ratios for OS between high vs. low CD169+ cell density per individual ROIs of pCRC and LM in CRC patients with synchronous and metachronous metastasis.

Groups		Synchronous		Metachronous
Regions	*N*	HR (95% CI), *p*‐value	*N*	HR (95% CI), *p*‐value
pTC	40 (74.1%)	**0.47 (0.23–0.94)**, **p** = 0.034	29 (69.0%)	2.04 (0.75–5.51), *p* = 0.161
pIM	39 (72.2%)	0.81 (0.39–1.70), *p* = 0.576	32 (76.2%)	0.44 (0.17–1.15), *p* = 0.096
pOM	41 (75.9%)	0.90 (0.43–1.85), *p* = 0.766	32 (76.2%)	0.46 (0.19–1.09), *p* = 0.078
pPT	40 (74.1%)	0.80 (0.39–1.65), *p* = 0.545	31 (73.8%)	0.67 (0.27–1.64), *p* = 0.377
mTC	36 (65.5%)	0.78 (0.41–1.48), *p* = 0.450	27 (61.4%)	1.55 (0.66–3.63), *p* = 0.310
mIM	40 (76.9%)	0.96 (0.43–2.11), *p* = 0.911	32 (74.4%)	1.18 (0.46–3.02), *p* = 0.737
mOM	40 (75.5%)	1.75 (0.77–3.99), *p* = 0.180	31 (73.8%)	**3.60 (1.05–12.37)**, **p** = 0.042
mPT	40 (75.5%)	2.05 (0.93–4.48), *p* = 0.073	31 (73.8%)	1.72 (0.63–4.69), *p* = 0.292

*Note*: Bold values indicate statistical significance at the *p* < 0.05 level.

Abbreviations: AJCC, The American Joint Committee on Cancer; CEA, the carcinoembryonic antigen; CHT, chemotherapy; HR (95% CI), Hazard ratio (95% confidence interval) shows the relative risk compared with 1.00 for the low density; LM, liver metastasis; mIM, inner margin of LM; mOM, outer margin of LM; mPT, peritumor area of LM; MSI, microsatellite instability; mTC, tumor center of metastasis; N, number and proportion of patients with high cell density; NOS, not otherwise specified; OS, overall survival; pCRC, primary colorectal cancer; pIM, inner margin of pCRC; pOM, outer margin of pCRC; pPT, peritumor area of pCRC; pTC, tumor center of pCRC; R0, no tumor cells detected at the resection margin; R1, tumor cells present at the microscopic resection margin; ROI, region of interest; WT, wild type.

### 3.8. Associations Between Clinical and Pathological Variables and OS

In the metachronous group, FOLFOX therapy was the only clinical factor associated with improved OS following LM resection (Table S5).

### 3.9. Multivariable Survival Analysis

In the multivariable Cox model, the association between OS and CD169+ macrophage densities in the TC of pCRC and the PT of LM remained significant in the synchronous group. The corresponding association in metachronous LM was of borderline significance (Table S6); instead, the FOLFOX regimen was independently predictive of longer OS.

### 3.10. Internal Validation

To validate survival associations internally, we applied a bootstrapping technique (Table S7). Within the synchronous group, high CD169+ macrophage density in the PT of LM remained significantly associated with shorter OS.

### 3.11. Associations of CD169+ Macrophage Density With Chemotherapy Regimen and Timing

Significant associations were only observed in the synchronous group. Higher cell densities in the TC and IM of pCRC were found in patients who received chemotherapy after LM resection (Table S8). Lower cell densities in the PT of pCRC and IM and PT of LM were observed in patients treated with the FOLFOX regimen (Table S9).

## 4. Discussion

In this study, we investigated the spatial distribution and clinical significance of CD169+ macrophages across pCRC, adjacent NM, and LM using immunohistochemistry and digital image analysis. We further assessed the prognostic association of CD169+ macrophage density and its correlation with tumor‐infiltrating T cells in our patient cohort. In the synchronous group, a high density of CD169+ macrophages in the TC of pCRC was associated with a longer OS, whereas a high density in the PT of LM was associated with a shorter OS. The high density of CD169+ macrophages in the OM of metachronous LM was associated with poor survival. These findings demonstrate that the prognostic associations of CD169+ macrophage distribution are context‐dependent.

### 4.1. CD169+ Macrophages are More Abundant in NM Than TC of pCRC

We observed significantly smaller densities of CD169+ macrophages in the TC of pCRC compared to the NM, which is supported by the literature [34]. This disparity may reflect the immunosuppressive remodeling that occurs within the tumor core, where hypoxia, lactic acid accumulation, and tumor‐derived cytokines (e.g., IL‐10 and TGF‐β) can suppress macrophage recruitment and polarize them toward the M2 phenotype [18, 35, 36]. In addition, the relative abundance of CD169+ cells in NM may reflect a more immunologically active or preserved microenvironment, potentially primed to respond to tumor antigens emanating from the neoplastic tissue [11, 21]. In the NM, CD169+ macrophages are involved in antigen presentation, secretion of chemokines, and recruitment of monocytes [11, 16, 21].

Two groups reported results discordant with ours. Ohnishi et al. [26] provided a descriptive observation of an absence of CD169+ macrophages in CRC epithelium or stroma despite their presence in normal colon and in infectious colitis. Another study found a higher percentage of CD169+ cells in the CRC tissue compared to colon lamina propria in healthy subjects using flow cytometry on a small cohort [18], which may explain discordant results.

### 4.2. Higher Cell Density in Tumor Exterior Than Tumor Interior

We observed a distinct spatial patterning of CD169+ macrophages in pCRC and LM, with enrichment in the OM and PT over tumor cores in both sites, which is in alignment with our previous study on T‐cell subsets in the same cohort [28]. Lower cell density within the tumor core may reflect an immune exclusion phenomenon, potentially driven by tumor‐derived factors that inhibit immune cell infiltration or promote an immunosuppressive milieu [37]. Another possible explanation is the activity of cancer‐associated fibroblasts at the tumor‐invasive front, which promote extracellular matrix remodeling and create structural barriers that restrict immune cell infiltration into the tumor core [38]. Consistently, spatial mapping studies indicated a preferential enrichment of pan‐macrophages and M2‐macrophages at the tumor boundaries compared to intratumoral regions [39, 40].

### 4.3. Higher Cell Density in LM vs. pCRC

Notably, this distribution was especially prominent in synchronous LM, where peritumoral CD169+ cell density was significantly higher than that in primary tumors. This suggests a potential recruitment or expansion of CD169+ macrophages in the metastatic niche, possibly driven by tumor‐derived chemokines (e.g., CCL2 and CXCL12) [41, 42] or hepatic microenvironmental cues such as IFN‐α [43]. Another possible explanation is the contribution of resident liver macrophages, such as Kupffer cells, which also express CD169 [44]. Therefore, CD169+ cells identified in LM may represent a heterogeneous population including both TAMs and liver‐resident macrophages with distinct biological functions. Since our study relied on CD169 immunohistochemical staining without additional markers to distinguish these populations, the specific contribution and functional role of CD169+ macrophage subsets in LM require further investigation.

### 4.4. Favorable Prognostic Effect of High Cell Density in pCRC in Synchronous Group and Unfavorable Associations in LM

We propose that the favorable prognostic associations of CD169+ cells in pCRC reflect their capacity to initiate and support adaptive immune responses, consistent with previous studies [17, 45–47]. This assumption is reinforced by significant correlations observed between CD169+ macrophages and CD3+ and CD8+ T lymphocytes in our cohort. Mechanistically, CD169+ macrophages may exert this effect through the release of type‐I interferons, which promote the activation and proliferation of both innate and adaptive immune cells [48, 49]. Additionally, CD169+ macrophages may facilitate immune cell recruitment by producing chemokines such as CCL7, CCL12, and CXCL14, thereby enhancing antitumor immunity and potentially restricting early tumor dissemination [50]. These mechanisms are consistent with the improved survival observed in patients. Our findings align with previous reports demonstrating that CD169+ macrophages in tumor‐draining lymph nodes interact with CD8+ T cells and NK cells, contributing to favorable clinical outcomes in CRC [25, 26, 51].

However, despite their higher density in LM compared to pCRC, CD169+ cells in both synchronous and metachronous LM were linked to worse outcomes. This paradox may be explained by the immunosuppressive nature of the liver microenvironment, characterized by elevated expression of IL‐10 and TGF‐β, checkpoint molecules like PD‐L1 [52], and the presence of dysfunctional immune cells [53, 54]. These factors may neutralize the potential antitumor activity of CD169+ macrophages in the liver. In the context of metachronous LM, which develop months or years after the primary tumor resection, the initial immune activation may have diminished or been lost over time [55, 56]. Furthermore, within the hepatic microenvironment, CD169+ macrophages may undergo polarization toward an M2‐like phenotype, contributing to fibrosis and stromal remodeling that support metastatic progression [18, 57, 58].

Taken together, the contrasting prognostic roles of CD169+ cells in primary tumors vs. LM highlight their functional plasticity. As a distinct macrophage subset, CD169+ cells can exhibit both M1‐like (e.g., IL‐12‐producing) and M2‐like (e.g., IL‐10‐producing) features depending on local cues [16, 18, 19]. Their polarization and functional behavior appear to be shaped by the specific immunological context of the TME [36]. Functional consequences of interactions between CD169+ macrophages and other immune cells appear to be context‐ and time‐dependent [35], which may explain the paradoxical association between high CD169+ macrophage density in primary tumor and LM and prognosis.

### 4.5. Association Between Chemotherapy Regimen and CD169+ Macrophage Distribution

We observed lower CD169+ macrophage density in the TC of pCRC among patients from the synchronous group who received chemotherapy before liver surgery compared with those receiving it after liver surgery. We also found lower CD169+ macrophage density in both pCRC and LM among FOLFOX‐treated patients compared with those receiving other regimens. This finding is consistent with reported effects of oxaliplatin‐based chemotherapy on macrophages in CRC [59]; however, the underlying mechanisms require further instigation.

Notably, FOLFOX treatment becomes an independent predictor of OS in patients with metachronous LM in both univariable and multivariable analyses. Previous studies have shown that FOLFOX can modulate macrophage polarization from protumor (M2) to an antitumor (M1) phenotype [60], which may help account for its clinical impact.

### 4.6. CD169 vs. Other Subtypes of Macrophages in CRC

Similar to other macrophage subtypes, CD169+ macrophages predominated in the superficial layer of mucosa and additionally within peripheral lymphoid follicles [11, 61]. In both pCRC and LM, CD169+ macrophages were more frequently localized in lymphoid aggregates, whereas other macrophage subtypes were predominantly localized in the stroma [11]. Like other macrophage subtypes, CD169+ macrophages were less abundant in the tumor interior than in the tumor exterior [39, 40]. All of the above findings are consistent with the preferential localization of CD169+ macrophages in lymphoid organs [16, 17]. Along with CD68+ and CD163+ macrophages, in contrast to CD80+ TAMs, CD169+ macrophages predominated in LM compared with pCRC [11, 62], which may indicate their skewing towards an M2‐like phenotype.

The observed favorable prognostic associations of CD169+ macrophages in the synchronous group are in contrast with the effects of conventional TAM subsets in CRC, which are predominantly associated with immunosuppression, angiogenesis, and tumor progression [39, 40] CD169+ macrophages represent a specialized macrophage population preferentially localized to perifollicular and invasive‐margin immune niches, where they participate in antigen capture, cross‐presentation, and activation of adaptive antitumor immune responses [25, 46, 47, 63].

Adverse prognostic associations of CD169+ macrophages in metachronous LM contrast with data from another study, which found that high CD68+ macrophage infiltration specifically within LM is associated with better outcomes [64].

### 4.7. Strength, Limitations, and Future Directions

To the best of our knowledge, this is the first study to evaluate the distribution of CD169+ cells across NM, pCRC, and synchronous vs. metachronous LM, as well as the association between CD169+ macrophages in pCRC and LM with patient outcomes. Our findings emphasize the importance of protein‐level assessment using immunohistochemical methods, which reveal critical microanatomical heterogeneity.

However, this study has several limitations. First, its retrospective design and reliance on a single‐institution cohort of modest size limit the generalizability of our findings. Second, we acknowledge that this study involves exploratory analyses across multiple tissue compartments and ROIs; accordingly, we admit that 85.5% of our significant findings are expected to be true positives. Validation in larger, independent cohorts is warranted in the future. Another limitation is the heterogeneity of neoadjuvant and adjuvant anticancer treatments, which precluded a reliable assessment of the associations between treatment, its effects, and macrophage infiltration. A further limitation is that CD169 expression in LM may identify a heterogeneous myeloid population, including resident Kupffer cells and TAMs, which may have distinct biological functions. Future studies should incorporate single‐cell approaches, multiplex imaging, transcriptomic profiling, and mechanistic analyses. Finally, while whole‐exome sequencing of pCRC and LM in this cohort has recently been published [65], we are currently investigating how genetic alterations intersect with tumor‐infiltrating immune cell profiles.

## 5. Conclusion

In conclusion, CD169+ macrophages exhibit distinct spatial‐ and context‐dependent prognostic associations in CRC. While high CD169+ macrophage density in the TC of pCRC was linked to favorable survival in the synchronous group, increased density in both synchronous and metachronous LM was associated with adverse outcomes. These findings underscore the context‐dependent role of CD169+ macrophages during CRC progression and metastatic evolution and suggest that their prognostic significance is determined by both the anatomical niche and the timing of metastasis. The biological mechanisms underlying the associations observed in this study remain to be elucidated through functional investigations. Validation in larger, independent patient cohorts will be essential to confirm these findings and establish their broader clinical utility.

## Author Contributions

**Wenjing Ye**: writing – review and editing (equal), formal analysis (equal), software (equal), investigation (equal), writing – original draft (lead). **Sergii Pavlov and Esraa Ali**: data curation (equal), software (equal), formal analysis (equal), investigation (equal), writing – review and editing (equal). **Filip Ambrozkiewicz**: formal analysis (equal), visualization (equal), validation (equal). **Lenka Červenková**: data curation (equal), resources (equal), investigation (equal), methodology (equal), writing – review and editing (equal). **Ondřej Vyčítal**: data curation (equal), formal analysis (equal), methodology (equal), resources (equal), investigation (equal), writing – review and editing (equal). **Petr Hošek**: visualization (equal), writing – review and editing (equal). **Ondřej Daum**: conceptualization (equal), investigation (equal), methodology (equal), validation (equal), data curation (equal), writing – review and editing (equal). **Václav Liška**: funding acquisition (equal), conceptualization (equal), validation (equal), project administration (lead), resources (lead), writing – review and editing (equal). **Kari Hemminki**: conceptualization (lead), funding acquisition (lead), methodology (equal), project administration (equal), supervision (lead), validation (equal), writing – review and editing (equal). **Andriy Trailin**: conceptualization (lead), investigation (equal), methodology (lead), validation (lead), software (lead), formal analysis (equal), data curation (equal), supervision (equal), writing – review and editing (lead).

## Funding

This work was supported by the Ministry of Health of the Czech Republic (Grants AZV NU21‐03‐00506 and AZV NW24‐03‐00521) and the Cooperation Program, Research Area SURG, the Integration of Biomedical Research and Health Care in the Pilsen Metropolitan Area (cofunded by the European Union and by the State Budget of the Czech Republic, Grant CZ.02.01.01/00/23_021/0008828).

## Ethics Statement

This study was conducted in compliance with the ethical standards outlined in the Declaration of Helsinki (2013 version). Due to the retrospective design of the study, the long time after which the tissues were collected, and the advanced stage of the disease, informed consent could not be obtained. The need for informed consent was waived by the Ethics Committee of the Faculty of Medicine and the University Hospital in Pilsen. The study was approved by the Ethics Committee of the Faculty of Medicine and the University Hospital in Pilsen (300/2020, June 17, 2020).

## Conflicts of Interest

The authors declare no conflicts of interest.

## Supporting Information

Additional supporting information can be found online in the Supporting Information section.

## Supporting information


**Supporting Information** Table S1: The estimated probability of overall survival for patients in synchronous and metachronous groups in Kaplan–Meier analysis. *Note*: ^1^Estimated probability of outcomes (95% confidence interval). Table S2: Spearman’s correlation between density of CD169+ macrophages and CD3+ T cells in the pCRC and LM of the synchronous and metachronous groups. *Note:* Bold values indicate statistical significance at the *p* < 0.05 level. Abbreviations: CI, confidence interval; LM, liver metastasis; mIM, inner margin of LM; mOM, outer margin of LM; mPT, peritumor area of LM; mTC, tumor center of metastasis; pCRC, primary colorectal cancer; pIM, inner margin of pCRC; pOM, outer margin of pCRC; pPT, peritumor area of pCRC; pTC, tumor center of pCRC. Table S3: Spearman’s correlation between density of CD169+ macrophages and CD8+ T cells in the pCRC and LM of the synchronous and metachronous groups. *Note:* Bold values indicate statistical significance at the *p* < 0.05 level. Abbreviations: CI, confidence interval; LM, liver metastasis; mOM, outer margin of LM; mPT, peritumor area of LM; pCRC, primary colorectal cancer; pIM, inner margin of pCRC; pOM, outer margin of pCRC; pPT, peritumor area of pCRC; pTC, tumor center of pCRC. Table S4: Cox regression analysis of density of CD169+ macrophage cell densities in NM and per individual ROIs of pCRC and LM as continuous variables for OS in CRC patients with the synchronous and metachronous liver metastases. *Note*: Bold values indicate statistical significance at the *p* < 0.05 level. Abbreviations: HR (95% CI), hazard ratio (95% confidence interval) shows the relative risk compared with 1.00 for the low density; LM, liver metastasis; mIM, inner margin in LM; mOM, outer margin in LM; mPT, peritumor area in LM; mTC, tumor center in metastasis; NM, nontumor mucosa; OS, overall survival; pCRC, primary colorectal cancer; pIM, inner margin in pCRC; pOM, outer margin in pCRC; pPT, peritumor area in pCRC; pTC, tumor center in pCRC; ROI, region of interest. Table S5: Associations of clinical and pathology variables in pCRC and LM with OS (univariable analysis) in the synchronous and metachronous groups. *Note*: Bold values indicate statistical significance at the *p* < 0.05 level. Median age: synchronous = 62, metachronous = 64; median size for primary tumor: synchronous = 4.3 cm, metachronous = 3.5 cm; median size of liver metastasis: synchronous = 2.0 cm, metachronous = 2.7 cm. Abbreviations: CHT, chemotherapy; CI, confidence interval; CRC, colorectal cancer; HR, hazard ratio; LM, liver metastases; OS, overall survival; pCRC, primary colorectal cancer. Table S6: Densities of CD169+ macrophage cells associated with overall survival (multivariable analysis) in CRC patients with synchronous and metachronous LM. *Note*: Bold values indicate statistical significance at the *p* < 0.05 level. Abbreviations: CHT, chemotherapy; LM, liver metastasis; LS, liver surgery; mOM, outer margin of LM; pCRC, primary colorectal cancer; pTC, tumor center of pCRC. Table S7: Cox hazard ratios and bootstrapped 95% confidence intervals for internal validations. *Note*: Bold values indicate statistical significance at the *p* < 0.05 level. Abbreviations: HR (95% CI), hazard ratio (95% confidence interval) shows the relative risk compared with 1.00 for the low density; LM, liver metastasis; mIM, Inner margin in LM; mOM, outer margin in LM; mPT, peritumor area in LM; mTC, tumor center in metastasis; NM, nontumor mucosa; pCRC, primary colorectal cancer; pIM, inner margin in pCRC; pOM, outer margin in pCRC; pPT, peritumor area in pCRC; pTC, tumor center in pCRC. Table S8: Association between CD169+ macrophage cell densities and chemotherapy before and after liver resection in CRC patients with synchronous and metachronous metastases. *Note*: ^1^Median (min–max) cell densities,  ^∗^ *p* < 0.05. Abbreviations: CHT, chemotherapy; CRC, colorectal cancer; IM, inner margin; LM, liver metastases; OM, outer margin; pCRC, primary colorectal cancer; PT, peritumor zone; TC, tumor center. Table S9: Association between CD169+ macrophage cell densities and FOLFOX‐based chemotherapy vs. other in CRC patients with synchronous and metachronous metastases. *Note*: ^1^Median (min–max) cell densities,  ^∗^ *p* < 0.05. Abbreviations: IM, inner margin; LM, liver metastases; OM, outer margin; pCRC, primary colorectal cancer; PT, peritumor zone; TC, tumor center.

## Data Availability

The data that support the findings of this study are available from the corresponding author (Andriy.Trailin@lfp.cuni.cz) upon reasonable request.
