# Supplementary material for: Spatial Profiling and Prognostic Role of CD169+ Macrophages in Colorectal Cancer From Adjacent Nontumor Mucosa to Liver Metastasis
Source: J Immunol Res. 2026 Sep 29;2026:7305785. doi: 10.1155/jimr/7305785 (PMC13624539; doi:10.1155/jimr/7305785)
Supplement: Supplementary file 1 [file JIMR-2026-7305785-s001.docx]

Table S1. The estimated probability of overall survival for patients in synchronous and metachronous groups in Kaplan-Meier analysis.

|  | **1y** | **3y** | **5y** |
| --- | --- | --- | --- |
| **Synchronous**  **Metachronous** | 87.2% (78.3%-96.1%)^1^  93.2% (85.7%-100.0%) | 51.9% (38.3%-65.5%)  62.6% (47.7%-77.5%) | 31.3% (17.9%-44.8%)  34.8% (17.0%–52.7%) |

Notes: ^1^ estimated probability of outcomes (95% confidence interval)

Table S2. Spearman’s correlation between densities of CD169+ macrophages and CD3+ T cells in the pCRC and LM of synchronous and metachronous group.

|  | **Synchronous** | | **Metachronous** | |
| --- | --- | --- | --- | --- |
|  | **rho** | **95% CI, *P* - value** | **rho** | **95% CI, *P* - value** |
| CD169 pTC vs CD3 pTC | 0.05 | (-0.23 to 0.32), *P* = 0.73 | 0.23 | (-0.05 to 0.48), *P* = 0.10 |
| CD169 pIM vs CD3 pIM | 0.04 | (-0.24 to 0.31), *P* = 0.76 | 0.25 | (-0.033 to 0.50), *P* = 0.07 |
| CD169 pOM vs CD3 pOM | -0.16 | (-0.42 to 0.12), *P* = 0.24 | -0.13 | (-0.40 to 0.15), *P* = 0.35 |
| CD169 pPT vs CD3 pPT | -0.38 | (-0.60 to -0.12), ***P* = 0.004** | -0.12 | (-0.39 to 0.17), *P* = 0.40 |
| CD169 mTC vs CD3 mTC | 0.38 | (0.08 to 0.62), ***P* = 0.012** | -0.002 | (-0.31 to 0.31), *P* = 0.99 |
| CD169 mIM vs CD3 mIM | 0.43 | (0.14 to 0.65), ***P* = 0.005** | 0.08 | (-0.24 to 0.38), *P* = 0.62 |
| CD169 mOM vs CD3 mOM | 0.17 | ( -0.15 to 0.46), *P* = 0.27 | 0.19 | (-0.13 to 0.47), *P* = 0.23 |
| CD169 mPT vs CD3 mPT | 0.16 | (-0.16 to 0.45), *P* = 0.32 | -0.26 | (-0.53 to 0.063), *P* = 0.10 |

Notes: Bold values indicate statistical significance at the *P* < 0.05 level. Abbreviations: pCRC: primary colorectal cancer. LM: liver metastasis. pTC: tumor center of pCRC; pIM: inner margin of pCRC; pOM: outer margin of pCRC; pPT: peritumor area of pCRC; mTC: tumor center of metastasis; mIM: inner margin of LM; mOM: outer margin of LM; mPT: peritumor area of LM; CI: confidence interval.

Table S3. Spearman’s correlation between densities of CD169+ macrophages and CD8+ T cells in the pCRC and LM of synchronous and metachronous group.

|  | **Synchronous** | | **Metachronous** | |
| --- | --- | --- | --- | --- |
|  | **rho** | **95% CI, *P* - value** | **rho** | **95% CI, *P* - value** |
| CD169 pTC vs CD8 pTC | 0.19 | (-0.09 to 0.44), *P* = 0.18 | 0.37 | (0.07 to 0.61), ***P* = 0.016** |
| CD169 pIM vs CD8 pIM | 0.26 | (-0.02 to 0.50), *P* = 0.06 | 0.44 | (0.15 to 0.66), ***P* = 0.004** |
| CD169 pOM vs CD8 pOM | 0.12 | (-0.1624 to 0.38), *P* = 0.39 | 0.11 | (-0.21 to 0.41), *P* = 0.50 |
| CD169 pPT vs CD8 pPT | -0.14 | (-0.3964 to 0.14), *P* = 0.33 | 0.06 | (-0.26 to 0.36), *P* = 0.73 |
| CD169 mTC vs CD8 mTC | 0.28 | (-0.002 to 0.52), ***P* = 0.045** | 0.13 | (-0.19 to 0.42), *P* = 0.42 |
| CD169 mIM vs CD8 mIM | 0.28 | (0.0002 to 0.52), ***P* = 0.044** | 0.16 | (-0.16 to 0.45), *P* = 0.30 |
| CD169 mOM vs CD8 mOM | -0.03 | (-0.30 to 0.26), *P* = 0.85 | 0.14 | (-0.18 to 0.43), *P* = 0.37 |
| CD169 mPT vs CD8 mPT | -0.21 | (-0.46 to 0.07), *P* = 0.13 | -0.14 | (-0.44 to 0.18), *P* = 0.37 |

Notes: Bold values indicate statistical significance at the *P* < 0.05 level.

Abbreviations: pCRC: primary colorectal cancer. LM: liver metastasis. pTC: tumor center of pCRC; pIM: inner margin of pCRC; pOM: outer margin of pCRC; pPT: peritumor area of pCRC; mTC: tumor center of metastasis; mIM: inner margin of LM; mOM: outer margin of LM; mPT: peritumor area of LM; CI: confidence interval.

Table S4. Cox regression analysis of density of CD169+ macrophage cell density in NM and per individual ROIs of pCRC and LM as continuous variables for OS in CRC patients with synchronous and metachronous liver metastases.

|  | **Synchronous** | | **Metachronous** | |
| --- | --- | --- | --- | --- |
|  | **HR** | **95% CI, *P* - value** | **HR** | **95% CI, *P* - value** |
| NM | 1.0001 | (0.9982-1.0019), *P* = 0.950 | 1.0010 | (0.9992-1.0029), *P* = 0.267 |
| pTC | 1.0002 | (0.9940-1.0065), *P* = 0.939 | 0.9963 | (0.9868-1.0058), *P* = 0.443 |
| pIM | 0.9980 | (0.9914-1.0046), *P* = 0.548 | 0.9975 | (0.9919-1.0031), *P* = 0.382 |
| pOM | 1.0002 | (0.9980-1.0023), *P* = 0.880 | 0.9991 | (0.9963-1.0019), *P* = 0.517 |
| pPT | 1.0003 | (0.9988-1.0018), *P* = 0.696 | 0.9997 | (0.9973-1.0020), *P* = 0.779 |
| mTC | 1.0008 | (0.9931-1.0086), *P* = 0.834 | 0.9833 | (0.9614-1.0056), *P* = 0.141 |
| mIM | 1.0006 | (0.9953-1.0059), *P* = 0.822 | 0.9756 | (0.9366-1.0162), *P* = 0.234 |
| mOM | 1.0010 | (0.9996-1.0025), *P* = 0.161 | 1.0008 | (0.9983-1.0033), *P* = 0.514 |
| mPT | 1.0010 | (1.0000-1.0020), ***P* = 0.045** | 1.0006 | (0.9990-1.0023), *P* = 0.460 |

Notes: Bold values indicate statistical significance at the *P* < 0.05 level.

Abbreviations: NM: non-tumor mucosa; pCRC: primary colorectal cancer; LM: liver metastasis; OS: Overall survival. ROI: region of interest. pCRC: primary colorectal cancer. pTC: Tumor center in pCRC; pIM: Inner margin in pCRC; pOM: Outer margin in pCRC; pPT: Peritumor area in pCRC; mTC: Tumor center in metastasis; mIM: Inner margin in LM; mOM: Outer margin in LM; mPT: Peritumor area in LM. HR (95% CI): Hazard ratio (95% confidence interval) shows the relative risk compared with 1.00 for the low density.

Table S5. Associations of clinical and pathology variables in pCRC and LM with OS (univariable analysis) in synchronous and metachronous group.

| Variables | Group | Synchronous | | Metachronous | |
| --- | --- | --- | --- | --- | --- |
|  |  | N (%) | HR (95% CI), *P* - value | N (%) | HR (95% CI), *P* - value |
| Gender | male | 34 (61.8) | 1.00 | 29 (65.9) | 1.00 |
|  | female | 21 (38.2) | 1.55 (0.82-2.93), *P* = 0.18 | 15 (34.1) | 1.55 (0.68-3.54), P = 0.30 |
| Age | under median | 30 (54.5) | 1.00 | 19 (43.2) | 1.00 |
|  | above median | 25 (45.5) | 0.61 (0.33-1.15), *P* = 0.13 | 25 (56.8) | 0.78 (0.34-1.79), *P* = 0.51 |
| Size of pCRC | under median | 27 (49.1) | 1.00 | 22 (50.0) | 1.00 |
|  | above median | 28 (50.9) | 0.76 (0.41-1.44), *P* = 0.40 | 22 (50.0) | 1.21 (0.53-2.76), *P* = 0.66 |
| Grade of pCRC | 1 | 9 (19.1) | 1.00 | 14 (31.8) | 1.00 |
|  | 2 | 32 (68.1) | 1.48 (0.59-3.72), *P* = 0.41 | 26 (59.1) | 0.84 (0.35-2.03), *P* = 0.70 |
|  | 3 | 6 (12.8) | 1.32 (0.39-4.54), *P* = 0.66 | 4 (9.1) | 1.41 (0.30-6.73), *P* = 0.67 |
| Sidedness | Right | 12 (21.8) | 1.00 | 8 (18.2) | 1.00 |
|  | Left | 43 (78.2) | 0.96 (0.45-2.02), *P* = 0.90 | 36 (81.8) | 0.50 (0.19-1.31), *P* = 0.16 |
| N grade | 0 | 13 (23.6) | 1.00 | 13 (29.5) | 1.00 |
|  | 1 | 22 (40.0) | 1.22 (0.56-2.64), *P* = 0.62 | 16 (36.4) | 0.93 (0.37-2.35), *P* = 0.88 |
| Median size of LM | 2 | 20 (36.4) | 1.50 (0.68-3.35), *P* = 0.32 | 15 (34.1) | 0.42 (0.14-1.30), *P* = 0.13 |
|  | under median | 27 (49.1) | 1.00 | 22 (50.0) | 1.00 |
|  | above median | 28 (50.9) | 0.77 (0.42-1.42), *P* = 0.40 | 22 (50.0) | 0.67 (0.30-1.52), *P* = 0.34 |
| Number of LM | 1 | 25 (46.3) | 1.00 | 25 (58.1) | 1.00 |
|  | >1 | 29 (53.7) | 1.60 (0.84-3.07), *P* = 0.16 | 18 (41.9) | 1.87 (0.83-4.18), *P* = 0.13 |
| Grade of LM | 1 | 21 (39.6) | 1.00 | 18 (42.9) | 1.00 |
|  | 2 | 32 (60.4) | 0.85 (0.44-1.65), *P* = 0.64 | 24 (57.1) | 1.01 (0.45-2.31), *P* = 0.97 |
| Margin of LM | R0 | 40 (76.9) | 1.00 | 29 (69.0) | 1.00 |
|  | R1 | 12 (23.1) | 0.97 (0.46-2.05), *P* = 0.93 | 13 (31.0) | 1.61 (0.70-3.71), *P* = 0.27 |
| CHT regimen | Other | 24 (43.6) | 1.00 | 28 (63.6) | 1.00 |
|  | FOLFOX | 31 (56.4) | 1.38 (0.72-2.63), *P* = 0.33 | 16 (36.4) | 0.36 (0.13-0.97), ***P* = 0.04** |
| Use of targeted therapy | No | 29 (59.2) | 1.00 | 30 (75.0) | 1.00 |
|  | Yes | 20 (40.8) | 1.03 (0.51-2.09), *P* = 0.93 | 10 (25.0) | 0.93 (0.36-2.40), *P* = 0.90 |

Notes: Bold values indicate statistical significance at the *P* < 0.05 level. Median age: synchronous = 62, metachronous = 64; median size for primary tumor: synchronous = 4.3 cm, metachronous = 3.5 cm; median size of liver metastasis: synchronous = 2.0 cm, metachronous = 2.7 cm.

Abbreviations: pCRC: primary colorectal cancer; LM: liver metastases; CRC: colorectal cancer; HR: hazard ratio; CI: confidence interval; OS: overall survival; CHT: chemotherapy.

Table S6. Densities of CD169+ macrophage cells associated with overall survival (multivariable analysis) in CRC patients with synchronous and metachronous LM.

| **Variables** | **Group** | **N (%)** | **HR (95% CI), *P* - value** |
| --- | --- | --- | --- |
| **Synchronous** |  |  |  |
| CD169 pTC | Low  High | 14 (25.9)  40 (74.1) | -  0.40 (0.18-0.88), ***P* = 0.023** |
| Size of LM | Under median | 27 (49.1) | - |
|  | Above median | 28 (50.9) | 0.86 (0.43-1.70), *P* = 0.66 |
| CHT regimen | Other | 24 (43.6) | - |
|  | FOLFOX | 31 (56.4) | 1.12 (0.56-2.28), *P* = 0.74 |
| Timing of chemotherapy | After LS | 12 (21.8) | - |
|  | Before LS | 37 (67.3) | 0.79 (0.33-1.90), *P* = 0.60 |
| CD169 mPT | Low  High | 13 (24.5)  40 (75.5) | -  2.66 (1.16-6.11), ***P* = 0.021** |
| Size of LM | Under median | 27 (49.1) | - |
|  | Above median | 28 (50.9) | 0.99 (0.51-1.91), *P* = 0.97 |
| CHT regimen | Other | 24 (43.6) | - |
|  | FOLFOX | 31 (56.4) | 1.40 (0.70-2.82), *P* = 0.35 |
| Timing of chemotherapy | After LS  Before LS | 12 (21.8)  37 (67.3) | -  0.66 (0.29-1.48), P = 0.31 |
| **Metachronous** |  |  |  |
| CD169 mOM | Low  High | 11 (26.2)  31 (73.8) | 3.55 (0.92-13.71), *P* = 0.07 |
| Size of LM | Under median | 22 (50.0) | - |
|  | Above median | 22 (50.0) | 0.81 (0.33-1.99), *P* = 0.65 |
| CHT regimen | Other | 28 (63.6) | - |
|  | FOLFOX | 16 (36.4) | 0.29 (0.10-0.87), ***P* = 0.027** |
| Timing of chemotherapy | After LS | 11 (25.0) | - |
|  | Before LS | 32 (72.7) | 0.88 (0.35-2.17), *P* = 0.77 |

Notes: Bold values indicate statistical significance at the *P* < 0.05 level.

Abbreviations: pCRC: primary colorectal cancer. LM: liver metastasis. pTC: tumor center of pCRC; mOM: outer margin of LM; CHT: chemotherapy; LS: liver surgery.

Table S7. Cox hazard ratios and bootstrapped 95% confidence intervals internal validations.

|  | **Synchronous** | | **Metachronous** | |
| --- | --- | --- | --- | --- |
|  | **HR** | **95% CI** | **HR** | **95% CI** |
| NM | 1.0001 | 0.9977-1.0024 | 1.0010 | 0.9986-1.0027 |
| pTC | 1.0002 | 0.9928-1.0091 | 0.9963 | 0.9814-1.0124 |
| pIM | 0.9980 | 0.9928-1.0052 | 0.9975 | 0.9704-1.0177 |
| pOM | 1.0002 | 0.9975-1.0026 | 0.9991 | 0.9840-1.0064 |
| pPT | 1.0003 | 0.9980-1.0022 | 0.9997 | 0.9925-1.0037 |
| mTC | 1.0008 | 0.9941-1.0090 | 0.9833 | 0.9581-1.0122 |
| mIM | 1.0006 | 0.9921-1.0110 | 0.9756 | 0.9393-1.0162 |
| mOM | 1.0010 | 0.9992-1.0030 | 1.0008 | 0.9961-1.0033 |
| mPT | 1.0010 | **1.0000-1.0020** | 1.0006 | 0.9982-1.0022 |

Note: Bold values indicate statistical significance at the *P* < 0.05 level.

Abbreviations: NM: non-tumor mucosa; pCRC: primary colorectal cancer; LM: liver metastasis; pCRC: primary colorectal cancer. pTC: Tumor center in pCRC; pIM: Inner margin in pCRC; pOM: Outer margin in pCRC; pPT: Peritumor area in pCRC; mTC: Tumor center in metastasis; mIM: Inner margin in LM; mOM: Outer margin in LM; mPT: Peritumor area in LM. HR (95% CI): Hazard ratio (95% confidence interval) shows the relative risk compared with 1.00 for the low density.

Table S8. Association between CD169+ macrophage cell densities and chemotherapy before and after liver resection in CRC patients with synchronous and metachronous metastases.

| **Variables** | **Cell type and location** | **Synchronous group (37 patients before and 12 after)** | | | | **Metachronous group (11 patients before and 32 after)** | | | |
| --- | --- | --- | --- | --- | --- | --- | --- | --- | --- |
|  |  | **TC** | **IM** | **OM** | **PT** | **TC** | **IM** | **OM** | **PT** |
| CHT before liver surgery  CHT after liver surgery | CD169 pCRC  CD169 LM  CD169 pCRC  CD169 LM | 16 (0-188)^1^*  8 (0-142)  26 (7-250)  5 (1-156) | 10 (0-244) *  4 (0-278)  48 (5-180)  6 (0-113) | 39 (2-658)  99 (3-865)  109 (6-415)  106 (3-817) | 105 (4-932)  218 (5-1395)  177 (17-373)  173 (16-974) | 5 (2-85)  5 (1-236)  8 (0-332)  2 (0-327) | 3 (1-60)  9 (1-63)  5 (0-676)  3 (0-298) | 32 (2-155)  57 (5-858)  52 (0-1140)  61 (2-666) | 133 (1-240)  108 (15-1256)  116 (3-945)  165 (3-912) |

Note: ^1^ median (min-max) cell densities, **P* < 0.05.

Abbreviations: CRC: colorectal cancer; pCRC: primary colorectal cancer; LM: liver metastases; TC: tumor center; IM: inner margin; OM: outer margin; PT: peritumor zone; CHT: chemotherapy.

Table S9. Association between CD169+ macrophage cell densities and FOLFOX-based chemotherapy versus other in CRC patients with synchronous and metachronous metastases

| Chemotherapy  regimen | Cell type and location | Synchronous (31 patients FOLFOX and 18 other) | | | | Metachronous (16 patients FOLFOX and 23 other) | | | |
| --- | --- | --- | --- | --- | --- | --- | --- | --- | --- |
|  |  | TC | IM | OM | PT | TC | IM | OM | PT |
| FOLFOX  Other | CD169 pCRC  CD169 LM  CD169 pCRC  CD169 LM | 16 (0-250)^1^  4 (0-130)  19 (0-188)  7 (1-156) | 10 (0-160)  2 (0-103) *  10 (0-244)  8 (0-278) | 28 (2-415)  89 (3-397)  101 (2-658)  124 (3-865) | 98 (4-486) *  160 (5-751) *  198 (8-932)  266 (16-1395) | 8 (1-332)  3 (0-327)  6 (0-85)  3 (0-236) | 5 (1-676)  5 (1-298)  4 (0-264)  3 (0-90) | 50 (0-1140)  30 (2-205)  42 (1-337)  61 (2-858) | 121 (5-945)  68 (3-516)  114 (1-695)  168 (6-1256) |

Note: ^1^ median (min-max) cell densities, **P* < 0.05

Abbreviations: pCRC: primary colorectal cancer; LM: liver metastases; TC: tumor center; IM: inner margin; OM: outer margin; PT: peritumor zone.
